# Label-free testing strategy to evaluate packed red blood cell quality before transfusion to leukemia patients

**DOI:** 10.1038/s41598-022-26309-5

**Published:** 2022-12-17

**Authors:** Jakub Dybas, Aleksandra Wajda, Fatih Celal Alcicek, Magdalena Kaczmarska, Katarzyna Bulat, Ewa Szczesny-Malysiak, Agnieszka Martyna, David Perez-Guaita, Tomasz Sacha, Katarzyna M. Marzec

**Affiliations:** 1 Jagiellonian Center for Experimental Therapeutics, Jagiellonian University, 14 Bobrzyskiego St., 30-348 Krakow, Poland; 2 Faculty of Chemistry, Jagiellonian University, 2 Gronostajowa St., 30-387 Krakow, Poland; 3 Lukasiewicz Research Network, Krakow Institute of Technology, 73 Zakopiaska St., 30-418 Krakow, Poland; 4 Forensic Chemistry Research Group, University of Silesia in Katowice, 9 Szkolna St., 40-006 Katowice, Poland; 5 Department of Analytical Chemistry, University of Valancia, Dr. Moliner 50, Burjassot, Spain; 6 Chair of Haematology, Faculty of Medicine, Jagiellonian University Medical College, 12 Sw. Anny St., 30-008 Krakow, Poland; 7 Department of Haematology, Jagiellonian University Hospital, 2 Jakubowskiego St., 30-688 Krakow, Poland

**Keywords:** Biophysical chemistry, Lipids, Proteins, Applied optics, Optical physics, Optical spectroscopy, Raman spectroscopy, Predictive markers, Prognostic markers, Laboratory techniques and procedures, Membrane biophysics, Cell death, Haematological diseases, Biochemistry, Biophysics, Cell biology, Chemical biology, Physiology, Biomarkers, Health care, Medical research, Chemistry, Optics and photonics, Physics

## Abstract

Patients worldwide require therapeutic transfusions of packed red blood cells (pRBCs), which is applied to the high-risk patients who need periodic transfusions due to leukemia, lymphoma, myeloma and other blood diseases or disorders. Contrary to the general hospital population where the transfusions are carried out mainly for healthy trauma patients, in case of high-risk patients the proper quality of pRBCs is crucial. This leads to an increased demand for efficient technology providing information on the pRBCs alterations deteriorating their quality. Here we present the design of an innovative, label-free, noninvasive, rapid Raman spectroscopy-based method for pRBCs quality evaluation, starting with the description of sample measurement and data analysis, through correlation of spectroscopic results with reference techniques' outcomes, and finishing with methodology verification and its application in clinical conditions. We have shown that Raman spectra collected from the pRBCs supernatant mixture with a proper chemometric analysis conducted for a minimum one ratio of integral intensities of the chosen Raman marker bands within the spectrum allow evaluation of the pRBC quality in a rapid, noninvasive, and free-label manner, without unsealing the pRBCs bag. Subsequently, spectroscopic data were compared with predefined reference values, either from pRBCs expiration or those defining the pRBCs quality, allowing to assess their utility for transfusion to patients with acute myeloid leukemia (AML) and lymphoblastic leukemia (ALL).

## Introduction

Packed red blood cells (pRBCs) refer to isolated human red blood cells (RBCs), stored for subsequent transfusion inside polyvinyl chloride (PVC) bags with addition of the preservative doped solution, called further the supernatant mixture (SM), used to prevent blood clotting and to maintain RBCs viability^1–3^. According to the guidelines, pRBCs can be stored at 4 ± 2 °C for up to 42 days^3^. During this time, RBCs undergo multiple biochemical and structural changes with intensity and rate depending on age, sex, and health condition of the blood donor^4–8^. These alterations can affect RBCs functionality and viability, impacting the blood transfer efficiency and potentially leading to the blood recipient health complications^3,9–12^. Tests carried out on pRBCs prior to the transfusion include the serological control of the ABO system and the D antigen of the Rh system, blood count, hematocrit (Hct), hemoglobin (Hb) level and the presence of the markers for the infectious agents transmitted by blood^13^. The only test evaluating the RBCs quality, conducted before the blood transfusion, is the control of hemolysis level based on smear analysis with threshold of 0.8% compared to the total weight of RBCs (according to the Council of Europe^14^). Nonetheless, evaluation of RBCs that did not undergo hemolysis, in the context of their functionality and viability, remains deficient due to the lack of a rapid, non-invasive, label-free diagnostic method.

Transfusion therapy could be required in various conditions where hematopoiesis is suppressed or impaired such as anemia, hemophilia, thalassemia, kidney disease, liver disease, cancer, sickle cell disease, etc.^15,16^. Patients with hematologic malignancies, especially the acute leukemias, typically have impaired hematopoiesis which most likely results in severe cytopenias that require blood or blood-related products transfusion. For instance, majority of patients with cancer live through anemia at some level which could arise from the malignancy itself and/or the cancer treatment^17^. PRBCs transfusion helps to increase blood counts which results in higher survival rates since anemia is associated with lower survival in patients with leukemia^18,19^. However, there are numerous works which suggest a potential relationship between the storage-related alterations in RBCs and transfusion-associated complications such as occlusion, multiorgan failure, and mortality^20–24^. Therefore, the need for a proper assessment method of pRBCs prior to transfusion is indisputable. For instance, RBCs lose their membrane content, e.g. lipids, through the storage which results in alterations in rheological properties, e.g. loss of deformability, that can cause occlusion in microcirculation, hence, insufficient oxygen delivery to tissues^23^. Moreover, reduced membrane integrity and increased fragility in RBCs through the storage may lead to hemolysis and release of hemoglobin^20^. Furthermore, RBCs rely on metabolizing glucose to lactate for energy supply since lack of mitochondria^25^. Lactic acidosis rarely occurs in hematologic malignancies, but it is associated with extremely poor prognosis, especially with leukemias^26^. Collectively, such markers could be used as an indicator of viability and functionality of pRBCs, and an appropriate pRBC quality evaluation prior to blood transfusion would likely minimize complications for the blood recipient and increase in transfusion efficiency^6,27,28^, especially in high-risk patients who need periodic transfusions due to leukemia, lymphoma, myeloma and other blood diseases or disorders^29–31^.

Herein, we present the design of the innovative label-free testing strategy for pRBCs quality evaluation prior to transfusion, its validation (N = 27), and verification in clinical environment for pRBCs transfusion to patients (N = 58) with acute myeloid (AML) or/and acute lymphoblastic leukemia (ALL). This innovative approach is based on label-free analysis of SM using Raman Spectroscopy (RS) – a rapid, non-invasive, and sensitive technique^32–35^. Other non-invasive and label-free methods allowing for blood or RBCs analysis were previously used in a certain approach such as prediction for blood transfusion purposes^36^ or detection of the sickle cells^37^, although not for the quality and functionality check of pRBCs prior to a blood transfusion. Our team comprehensively reviewed the advantages of Raman Spectroscopy in the analysis of RBCs with careful comparison to the classical and other spectroscopy techniques^38^. Moreover, the application of RS to study the RBCs and to evaluate the pRBCs quality^38–42^ was previously reported. Recently, Vardaki et al. also studied RBC condition based on the measurements of SM using Raman and SORS approaches, however, with focus mainly on lactates and glucose levels^43,44^, for the general hospital population of patients. Other works in the field were mainly concentrated on measurement of Hb alterations of RBCs utilizing a small group of samples^41,42,45^. The design presented in our work is based on the RS analysis of SM content carried out weekly during 8 weeks of storage for statistically significant number of pRBCs obtained from the female and male donors (N = 27). With a proper chemometric analysis, we are not only able to assess the extent of RBCs hemolysis^46^ but also evaluate the content of RBCs metabolites released to SM. To correlate the results obtained from SM analysis using the RS with the absolute concentrations of every SM component, the samples were evaluated using quantitative reference techniques. Simultaneously with RS, SM biochemical parameters, including total cholesterol, glucose, free iron ions, total content of lactic acid derivatives, and triglycerides, were assessed. In addition, to correlate the observed SM alterations with RBCs quality in pRBCs, the hemolysis level using flow cytometry was analyzed. For the first time, the RS approach allowing for the determination of spectroscopic reference values of pRBCs quality and to design our novel strategy, was validated in the clinical environment (N = 58).

In conclusion, we have shown that Raman spectra collected for SM with a proper chemometric analysis conducted for minimum one ratio of integral intensities of the chosen Raman marker bands allow to define the pRBCs quality in a rapid, non-invasive, free-label manner, without opening the pRBCs bag. The results of the analysis are compared to the predefined spectroscopic reference values, either from pRBCs expiration or ones specific to the pRBCs quality, to assess its utility for transfusion to AML and ALL patients. Moreover, there are various conditions such as hemophilia, thalassemia, sickle diseases, etc., that requires blood transfusion where the method presented herein would be useful to assess pRBCs quality rapidly prior to the transfusion.

## Material and methods

### Packed red blood cells (pRBCs) collection

Leukocyto-deplete (N = 24) and non-leukoreduced (N = 3) pRBCs containing (Saline, Adenine, Glucose, Mannitol) additive solution and a trace amount of CPD (Citrate, Phosphate, Dextrose) preservative were purchased from the Regional Center for Blood Donation and Hemotherapy in Krakow. Citrate–phosphate-dextrose (CPD) solution was composed from citric acid monohydrate (3.27 g/dm^3^, i.e.15.6 mM), sodium citrate dihydrate (26.3 g/dm^3^, i.e. 89.4 mM), sodium dihydrogen phosphate dihydrate (2.51 g/dm^3^, i.e. 16.1 mM), glucose monohydrate (25.5 g/dm^3^, i.e. 128.7 mM). pH was adjusted to 5.6 ± 0.3. Concentration of sodium ions is 285 mM. Saline-adenine-glucose-mannitol (SAGM) solution was composed from sodium chloride (8.77 g/dm^3^, i.e. 150.1 mM), mannitol (5.25 g/dm^3^, i.e. 28.8 mM), glucose monohydrate (9.00 g/dm^3^, i.e. 45.4 mM), adenine (0.17 g/dm^3^, i.e. 1.3 mM). Concentration of sodium ions was 150 mM. According to the principles outlined in the World Medical Association (WMA) Declaration of Helsinki as well as a Bioethical Commission of the Jagiellonian University bags were prepared from the whole blood donated by men N = 12, aged < 30 years (N = 3), 30–39 years (N = 4), > 40 years (N = 5) and women N = 12, aged < 30 years (N = 4), 30–39 years (N = 4), > 40 years (N = 4). All analyzes were carried out weekly throughout 6 weeks of pRBCs storage, while the eighth week’s measurements were designed as an additional time point exceeding pRBCs’ expiration date (42 days). Before each week’s measurements, pRBCs were properly mixed.

### Procedure of pRBC samples preparation and measurements

The long-term multimodal experiments were carried out weekly for 8 weeks for pRBCs samples. Each experimental day the following experimental protocol was followed:

1. The pRBCs storage bag was removed from 4 ± 2 °C fridge and gently mix for approximately 5 min.
2. 15 mL of pRBCs sample was acquired using single-use syringe (20 mL, Norm-Ject Luer, Henke Sass Wolf, Germany) equipped with single-use needle (size 0.90 × 40 mm, Sterican, B. Braun, UK, article code 4,657,519) and transferred into falcon tube (15 mL, Bionovo, Poland).
3. The puncture point on pRBCs storage bag was sealed with 2.5 × 3 cm slice of the medical adhesive tape (Safeline Fol, MercatorMedical, Poland) and the pRBCs storage bag was placed back in 4 ± 2 °C fridge.
4. As the control, the following reference techniques are employed for pRBCs samples:
  - Lactates level – assessed using lactometer Lactate Scout + portable analyzer (EKF Diagnostics, Germany). The 3 µL of the pRBCs sample was applied with a pipette on the single use The Lactate Scout sensors (XYZ) in accordance with the manufacturer’s instruction. The samples were tested three times and the average value was calculated.
5. Subsequently, the acquired pRBCs sample was centrifuged using SIGMA 3–18 K centrifuge (Polygen, Poland) in order to separate SM. First, sample was centrifuged at 500×*g*, RT, 10 min followed by careful removal of the supernatant and second centrifugation: 3000×*g*, RT, 10 min.
6. As the control reference technique for separated RBCs, flow cytometry was employed. Samples containing 1 µL of pRBCs were transferred to 5 mL round-bottom tubes (BD Falcon) filled with 100 µL of 0.09% NaCl containing 0.05% of nuclei stain- Hoechst 33,342 (Thermo Scientific cat. no. H3570), a mixture of mouse anti-human antibodies carrying fluorescent markers: CD45 – APC-Cy7 (common leukocyte antigen, 1:200, BD cat. no. 348815), CD71 – APC (antigen present on the RBC progenitors, 1:200, BD cat. no. 551374), CD47 – PE (integrin-associated protein, 1:200, BD cat. no. 556046). Additionally, extra 1 µL of pRBCs were transferred to separate set of round-bottom tubes containing 100 µL of Annexin V Binding Buffer diluted 1:10 in DPBS (no calcium, no magnesium, cat. No. 14190144, ThermoFisher Scientific, Waltham, USA) with Annexin V – FITC (a marker of apoptotic cells, 1:200, BD cat. no. 556547). The cells were incubated in the dark (30 min, RT) and diluted in 400 µL of PBS.
7. Collected supernatant after second centrifugation was carefully collected and transferred into Eppendorf tubes (2 mL, safe-lock type, Bionovo, Poland) ready for measurements:
  - Raman Spectroscopy (RS) – 50 µL of SM sample was transferred onto CaF_2_ glass slide (Raman Grade, Crystran, UK) and left for 60 min to dry. Subsequently, dried SM samples were measured with 488 and 785 nm excitation wavelengths in the single spectra mode with the methodological details described in the next section.
  - Biochemistry – 300–400 µL of SM was transferred into the sample cup (Pentra 400/ABX MIRA, Horiba ABX, France, cat. no. A11A01765) and used for determination of lactic acid, glucose, cholesterol, triglyceride and iron concentration the Horiba ABX reagents were needed (Lactic Acid—cat. no. A11A01721, Glucose HK CP—cat. no. A11A01667, Cholesterol CP—cat. no. A11A01634, Triglycerides CP—cat. no. A11A01640 and Iron CP cat. no. A11A01637, respectively). An ABX Penta 400 (Horiba Medical, Japan) biochemical analyzer was employed. Each sample was measured two times.

### Raman spectroscopy

In case of both excitation wavelengths, 488 and 785 nm, Raman spectra were carried out on WITec confocal Raman microscope CRM Alpha 300RSA + (WITec GMBH, Ulm, Germany) equipped with UHTS300 and Action 2300i spectrometers (WITec GMBH, Ulm, German) characterized by maximum quantum efficiency adjusted to VIS and NIR regions, respectively. The monochromators of the spectrometers are calibrated monthly with the use of the radiation spectrum from the calibration xenon lamp (UV light source, WITec GMBH, Ulm, German). The standard alignment procedure was performed daily prior to analysis with the use of the Raman scattering line produced by a silicon plate (520.5 cm^−1^).

### (1) 488 nm

The Raman scattering signal was generated by solid-state laser operating at 488 nm excitation wavelength and the laser power at the laser focus spot set on 3 mW using PM100D Handheld Digital Power Meter (ThorLabs, Ann Arbor, Michigan, USA) adjusted for this wavelength. The laser was coupled to the microscope by an optical fiber with a diameter of 50 μm and signal was collected by the Andor Newton DU970N-BV-353 CCD camera (Oxford Instruments, Abingdon, England) with 1600 × 200 active pixels and 16 × 16 µm pixel size. The CCD camera was thermoelectrically cooled to − 60 °C during the measurements. The 600 g/mm grating was used providing 3 cm^−1^ spectral resolution of the collected spectra.

### (2) 785 nm

The Raman scattering signal was generated by solid-state laser operating at 785 nm excitation wavelength and the laser power at the laser focus spot set on 80 mW using PM100D Handheld Digital Power Meter (ThorLabs, Ann Arbor, Michigan, USA) adjusted for this wavelength. The laser was coupled to the microscope by an optical fiber with a diameter of 100 μm and signal collected by the Andor iDUS DU401A-BR-DD-352 CCD camera (Oxford Instruments, Abingdon, England) with 1024 × 127 active pixels and 26 × 26 µm pixel size. The CCD camera was thermoelectrically cooled to − 60 °C during the measurements. The 300 g/mm grating was used providing 3 cm^−1^ spectral resolution of the collected spectra.

### RS spectra processing

All acquired Raman spectra were preprocessed, including cosmic rays removal and the frequency range of a data file cutting to obtain a certain frequency range of spectra (using WITec Software 5.0). Then, a vector normalization in the set spectral range (350–3100 cm^–1^ ) and offset correction were performed (using OPUS 7.2 software). Based on the Savitzky-Golay algorithm the spectra were smoothed (9 smoothing points) to decrease the superimposed noise interferences. Additionally, the spectra were baseline corrected using asymmetric least squares smoothing (OriginLab 2019 software). To evaluate the integral intensity ratios of the appropriate metabolites found in SM samples, OPUS 7.2 software and the A-type integration method (Applied integration method was based on calculation of the integral value represented as the area bounded by the band shape, abscissa and the wavenumbers limits defined as local minima of the given band) were applied. The following ranges of spectra were taken into consideration: 390–478, 472–578, 830–870, 870–910, 1520–1695, and 2867–2964 cm^–1^ to analyze the integral intensities of the chosen bands. The box plots were constructed using OriginLab 2019 software to graphically depict the representation of the numerical data and their distribution of statistical features.

### Procedure of SORS measurements

SORS measurements of SM in the PVC storage bag were carried out with the use of Resolve Raman Handheld Through-Barrier Identification System (Agilent Technologies, Inc., California, USA). Agilent’s Resolve analyzer utilizes an excitation wavelength of 830 nm with a maximum laser power of 475 mW. The spot size of the laser was approximately 2 mm in diameter. The equipment was calibrated before the measurements. SORS spectra of the samples were obtained with through-barrier mode and 5.5 mm offset position (or surface scan mode for PVC bag). The analyzer and pRBCs storage bag were placed stationary, in stable conditions and darkened room during the measurement. pRBCs were unmixed so that SM was separated from the RBCs. Each sample was tested four times at different points with 1 s integration time over 5 accumulations.

### Design of label-free testing strategy to evaluate pRBC quality

In the strategy presented herein, we propose multimodal approach to follow the changes during long-term storage of pRBC samples described in *Packed red blood cells (pRBCs) collection* section. The strategy was designed based on measurements carried out weekly throughout 6 weeks of pRBCs storage (according to the expiration date of pRBCs, i.e. 42 days). Additionally, samples were checked in the seventh- and eighth-week to acquire the additional time points, which exceeding the pRBCs’ expiration date. The insight into pRBC membrane biochemistry, physical and mechanical properties as well as nanoscale changes of pRBC membrane were presented in separate publications^1,5,47^. Herein, for the first time, we present results from detailed SM analysis using Raman Spectroscopy (RS) approach. Acquired data was correlated to the reference techniques which included: quantitative analysis of lactate, glucose, total free iron, triglycerides, cholesterol and RBCs apoptosis (which were provided by biochemical analysis of SM as well as flow cytometry – representative results are shown in Additional File 1: Figs. S1 and S2).

**Figure 1:**
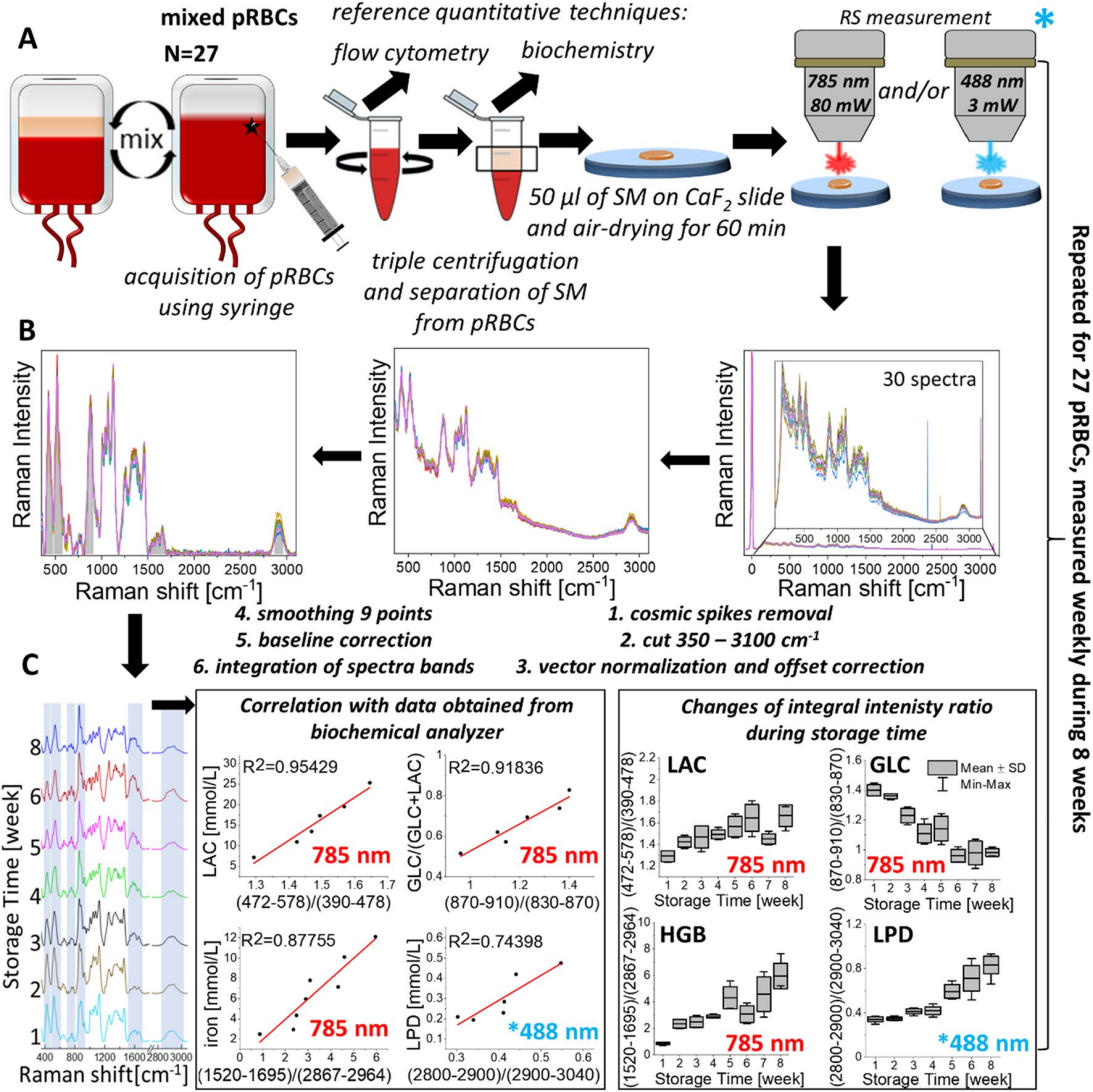
Schematic overview of the designed strategy. (**A**) pRBCs samples preparation and RS measurement intended for weekly SM analysis during period of 8 weeks in correlation to reference quantitative analysis to define the most sensitive and reliable RS spectral marker bands of pRBCs quality determination. The pRBC bags were mixed, subsequently, samples were aspirated using syringe directly through the PVC storage bag and then centrifuged in plastic tubes. Separated as upper fraction SM was aspirated and divided into two—first used for reference quantitative analysis and second for RS measurements. In the latter, 50 μ of SM was transferred into CaF_2_ slide and left to 60 min until completely dried. The RS measurements were carried out using WITec Alpha 300 with air-objective with 100×magnification (Olympus, MPlan, NA = 0,9). The spectra were recorded with 785 nm and 488 nm excitation wavelengths and the laser power at the laser spot approximately 130 mW and 3 mW, respectively. Thirty spectra were recorded from randomly chosen places within SM sample with the acquisition time of 3 s and number of accumulations of 10. All measurements were carried out weekly during period of 8 weeks of pRBCs storage (the term of validity is 42 days–6 weeks; the measurement after 7 and 8 weeks of pRBCs storage were carried out after their validity date). (**B**) RS spectra preprocessing procedure. Spectra preprocessing included removal of cosmic rays (using WITec Software 5.0), vector normalization in the whole spectral range (400–3050 cm^–1^, using OPUS 7.2 software) and additionally baseline corrected (using asymmetric least square method implemented in OriginLab 2019 software). (**C**) Definition of the most reliable in pRBCs quality evaluation ratios with the highest correlation to the reference techniques values. To assess the integral intensity ratios of the metabolites found in SM samples, OPUS 7.2 software and A-type integration method were used (i.e. the area above abscissa, restricted with the band shape and the frequency limits defined, suitable for integration of bands on baseline corrected spectra). Data obtained from RS were correlated with data obtained using reference techniques. Box charts constructed using OriginLab 2019 software were used to assess graphical representation of statistical features distribution of the most reliable markers of pRBCs quality including LAC, GLC, HGB and LPD.

During the design stage and the development of the strategy, (schematic overview is presented in Fig. 1) RS spectra (300 spectra for sample with the integration time of 3 s) of air-dried SM (N = 27) were collected using two different excitation wavelengths – 785 and 488 nm. The first excitation provided Raman spectra with greatly diminished impact of the resonance effect evoked by presence of the heme prosthetic group and thus allowed for observation of the bands related to the other components present in SM such as lactose and glucose. Moreover, the 785 nm-excited Raman spectra delivered more precise data on Hb concentration, keeping its relation with Raman intensity signal more linear. In turn, 488 nm laser line, was far better choice in analysis of the high wavenumber (2800–3100 cm^–1^) region allowing to define lipids to proteins ratio^48,49^. The fingerprint region of the Raman spectra recorded with 488 nm excitation is dominated by heme-related modes, what prevented from analysis of the other SM components ^38^. The bands of SM spectra were carefully preprocessed and assigned to the vibrational modes of distinct groups of compounds ^50,51^. The band assignments of the defined ranges of the pRBCs spectra were based on measured Raman spectra of the reference compounds: adenine, mannitol and glucose (which all are components of SAGM additive solution), SAGM additive solution used for pRBCs preparation, sodium lactate and free hemoglobin (which are main RBC metabolites^52,53^)—see Additional File 1: Figs. S3, S4 and Additional File 1: Table S1 for spectra of standard compounds and detailed band assignment.

The Raman spectrum of pRBCs after 1 week of storage is highly comparable with the SAGM spectrum, i.e. SAGM components including glucose, mannitol and adenine in approximate concentrations as found in SAGM. The Raman spectrum of pRBCs after 8 weeks of storage is comparable with the spectra of SAGM components with comparable concentrations of glucose, mannitol and adenine but including new components related to the pRBCs aging process and generation of RBC metabolites, including Hb and lactates^52,54^.

In addition, to further support the ability of the method to quantitate the selected components, the changes in relative intensities of chosen marker bands were correlated with the quantitative results obtained with the reference techniques (Additional File 1: Figs. S5–S8). The integral intensity (area under the band), reflecting the amount of the functional groups participating in the band origin, was calculated for each predefined marker band. Subsequently, the integral intensity is proportional to the concentration of the given compound and relates to vibrations of its characteristic functional groups. The chemometric analysis of as little as one ratio of such marker bands of the Raman spectrum, collected according to the developed method, was conducted based on evaluation of the marker band integral intensities or the chemometric analysis including mathematical operation, including machine learning algorithms, utilizing the given marker band spectral ranges.

The relationship between the Raman band intensities and the concentration of the analyzed compounds was investigated by using Partial Least Squares (PLS) method, with the generation of calibration sets to optimize the models and the evaluation of independent test sets for assessing the performance. Even though the prediction of the error for the multicomponent quantification was difficult due to the strong correlation among the parameter values, the results proved the PLS models were able to predict successfully the concentration of different parameters such as glucose and lactate (Relative Root Mean Square error of prediction < 6%). In Additional File 1: Fig. S9 we present the modeling process as well as details of the prediction versus actual values and the regression vectors. An even distribution of the points along the prediction line (Actual = Predicted) is shown in green. The Root Mean Square of Prediction (RMSEP) was found to be 5.5 mM and 6.5 mM for glucose and lactate, respectively. The relative, percent RMSEP, calculated by dividing the RMSEP by the average concentration value and multiplying by 100, was found to be 5% for glucose and 6.5% for lactate indicating high potential of Raman spectra data mining to calculate the actual concentration of the compounds tested related to evaluated clinical parameters. We have defined four ratios with the highest correlation to the reference techniques that were the most reliable in label-free SM analysis and can be treated as spectral determinants of pRBCs quality: increase in lactates (LAC) reflected by ratio (472–578)/(390–478) cm^–1^, decrease in glucose (GLC) reflected by ratio (870–910)/(830–870) cm^–1^, and increase in free hemoglobin (HGB) reflected by ratio (1520–1695)/(2857–2964) cm^–1^ – all obtained under 785 nm excitation, and increase in lipids (LPD) reflected by ratio (2800–2900)/(2900–3030) cm^–1^ obtained with 488 nm excitation. Furthermore, the regression vector indicates the bands responsible for the PLSR (Additional File 1: Fig. S10). When compared with the regions of the ratios used in the evaluation of the blood quality 870–910 cm^-1^/830–870 cm^-1^ for glucose and 472–578 cm^-1^/390–478 cm^-1^ for lactate, the region in the denominator always points to positive values of the regression vector, while the region in the nominator points to negative values providing quantitative potential of the spectra for clinical parameters and presenting evidence that the machine learning techniques can be applied to investigate the changes in concentration of these compounds over time.

The **LAC ratio** corresponds to the integral intensity of band in the range of 472–578 cm^–1^ to the integral intensity of the band in spectral range of 390–478 cm^–1^. The increase in lactate concentration with time of the pRBCs storage causes greater impact of lactates vibration found in 472–578 cm^–1^ spectral range compared to 390–478 cm^–1^ spectral range (band located at around 535 cm^–1^ is approximately three times more intense compared to the band at around 425 cm^–1^ in sodium lactate reference compound). The change in this ratio is correlated with the change in lactate concentration as observed with reference techniques. The line coefficient of determination (R^2^) was 0.83 on average indicating the accuracy of the analysis of integral intensity ratio of the given spectral ranges. The ratio of integral intensity of wagging vibrations of carboxylate ion (band at about 535 cm^–1^) to rocking vibrations of carboxylate ion (band at about 425 cm^–1^) increases with increase in lactic acid esters compared to lactic acid and lactate ion^55^. The content of lactic acid esters increases with time of the pRBCs storage indicating impairment of RBCs biochemical state.

The **GLC ratio** corresponds to the integral intensity of band in the range of 870–910 cm^–1^ to the integral intensity of band in the range of 830–870 cm^–1^. The decrease in GLC ratio corresponds to the decrease in glucose (G) concentration and the sum of decrease in glucose (G) and increase in lactates (L) [G/(G + L)]. This ratio again changes with time of the pRBCs storage and highly correlates with biochemical data (R^2^ > 0.95).

The **HGB ratio** corresponds to the integral intensity of band in the range of 1520–1695 cm^–1^ to the integral intensity of band in the range of 2867–2964 cm^–1^. The increase in HGB ratio over time of the pRBCs storage corresponds to an increase in Hb concentration. This ratio is correlated with concentration of the free iron ion in SM and the line coefficient of 0.93 on average.

The **LPD ratio** corresponds to the integral intensity of band in the range of 2800–2900 cm^–1^ to the integral intensity of band in the range of 2900–3040 cm^–1^. The LPD ratio increases with time of the pRBCs storage and corresponds to an increase in lipids/proteins ratio. This ratio was correlated with the increase of lipids obtained with biochemical analyzer and the line coefficient of 0.89 on average.

While evaluating all presented ratios allows for accurate analysis, each one of the described integral intensity ratios can be compared to the reference values to define the pRBCs quality independently.

### pRBCs samples preparation for verification of the designed strategy in clinical environment

Leukoreduction is intended to significantly reduce patient adverse reactions and pathogen transmission so all studied in this stage pRBCs were leukoreduced containing SAGM and CPD. They were purchased from the Regional Center for Blood Donation and Hemotherapy in Krakow and further transfer to the AML and ALL patients with the principles outlined in the World Medical Association (WMA) Declaration of Helsinki as well as a Bioethical Commission of the Jagiellonian University. The study was approved by the ethical approval of the Bioethical Commission of the Jagiellonian University No. 1072.6120.206.2019 of September 19, 2019 (with later extensions of the duration of the research) and the consent of the University Hospital in Krakow (CIT.060.02.2019) based on which the 1 mL of pRBCs were collected into Eppendorf, which are routinely transfused in the clinic to patients with acute myeloid and / or lymphoblastic. The collected fraction of pRBCs within 48 h were analyzed with the use of RS and biochemical parameters. The results were correlated with the set of clinical data for patients before and after the pRBCs transfusion for qualitative analysis assigned to each sample. The clinical data set analyzed for each patient before and after the transfusion included:

- a. Gender and age of the patient;
- b.The results of standard diagnostic tests performed in the hospital before and after the transfusion (within 24 h of the procedure), such as blood count, kidney function, inflammation and hemolysis;
- c. Results of non-standard tests (not performed routinely) performed in the hospital before and after transfusion (within 24 h of the procedure): lipid profile, CRP, serum creatinine with estimated GFR, uric acid, serum urea.

Informed consent was obtained from each patient whose diagnostic test results were used in this study.

## Results and discussion

### Definition of the RV-Res and RV-Exp values towards label-free testing strategy of pRBCs quality

The reference value of pRBCs expiration (RV-Exp) was defined in order to reject pRBCs unsuitable for transfusion. The RV-Exp value was determined independently for each ratio of integral band intensities described in *Materials and Method* section and was based on data collected using RS at 6th week of pRBCs storage for 27 pRBCs collected from both female (N = 12) and male (N = 15) donors. The LAC ratio higher than 2.0 (based on the value of 1.8 ± 0.2), the GLC ratio lower than 1.0 (based on the value of 0.9 ± 0.1), the HGB ratio higher than 6.0 (based on the value of 4.4 ± 1.6) and the LPD ratio higher than 0.7 (based on the value of 0.6 ± 0.1) were found to correlate to expired pRBCs based on the calculation of the average ratios obtained at 42nd day of storage. Based on our analysis those values indicate the threshold for the expired pRBCs in a rapid and accurate way.

**Figure 2:**
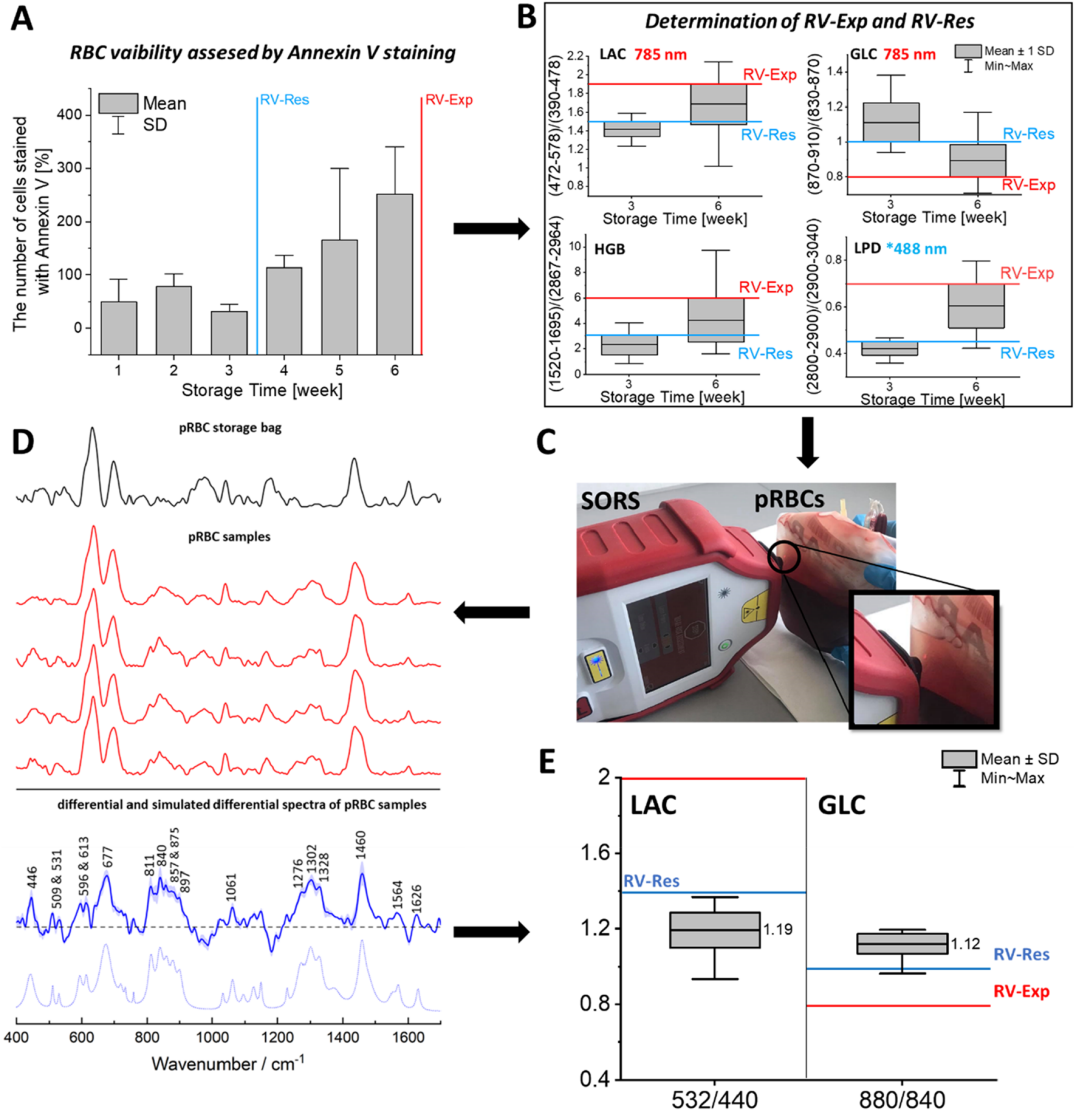
(**A**) RBCs hemolysis level observed for pRBCs evaluated with flow cytometry indicating the position of the threshold value of pRBCs quality (RV-Res) and reference value of pRBCs expiration (RV-Exp); (**B**) Determination of the RV-Res and RV-Exp values for LAC, GLC, HGB and LPD (N = 27) using RS; (**C**) SORS measurement carried out on SM via pRBCs bag; (**D**) The average SORS spectra of SM presented with the standard deviation (red solid line), differential spectrum (blue solid line) of the averaged SM spectra and the clean bag (black line) and simulated differential spectrum with the positive peaks only (blue scattered line). (**E**) Evaluation of pRBCs quality based on SORS measurements, based on the comparison of the obtained average value of LAC and GLC (presented as boxed charts) with RV-Res and RV-Exp values, indicating that studied pRBCs is not expired and has adequate quality to be transfer to o patients with an increased risk of post-transfusion complications.

Reference values can be defined independently also for the more stringent or loose requirements of pRBCs quality depending on the patient risk of complication after transfusion. The restrict reference value of pRBCs quality (RV-Res) was defined to indicate the pRBCs with quality insufficient for transfusion to patients with an increased risk of post-transfusion complications. The RV-Res threshold was determined independently for each ratio of integral band intensities presented in the previous section based on data analysis carried out in the 3rd week of storage^56^, where the greatest increase in hemolysis was observed based on the results of flow cytometry, for 27 pRBCs collected from both female (N = 12) and male (N = 15) donor. The RV-Res were defined based on an average ratio observed at third week of storage due to the highest increase of apoptotic RBCs between third and fourth week of storage. The RV-Res of LAC was defined at 1.5 (based on the value of 1.4 ± 0.1), GLC was defined at 1.0 (based on the value of 1.1 ± 0.1), HGB was defined at 3.1 (based on the value of 2.3 ± 0.8) and LPD was defined at 0.4 (based on the value of 0.42 ± 0.02). The results and process of definition of RV-Exp and RV-Res are presented in Fig. 2A,B and Additional File 1: Figs. S11–S13.

Moreover, we have proven our strategy can be successfully performed with the handheld spatially-offset Raman Spectroscopy (SORS) device and the measurements can be carried out directly through the PVC storage bag in the blood donation centers or hospitals on the separated SM phase as presented in Fig. 2C–E. The RS spectra obtained from SM with the use of SORS can be processed to deliver the information about LAC, GLC, HGB and LPD values that can be compared with RV-Exp as well as RV-Res and would be indented as the point of use analysis of pRBCs for the transfusion in the high-risk patients.

### Application of the designed strategy in laboratory environment

The goal of our testing strategy is to determine if the pRBCs fulfil the reference ratio of expiration (RV-Exp) or the restrict reference value of pRBCs quality (RV-Res). Different approaches for the comparison between values of ratios measured for specific pRBCs with RV-Exp and RV-Res can be applied, depending on applied laser excitation and the number of ratios considered during evaluation.

The content of SM at the time of pRBCs analysis depends strongly on the donor profile^57^. In some pRBCs we observe a high hemolyzes being present in the third week of storage while the lactate levels are low. Sometimes the situation is opposite and even if the hemolysis level is low, the level of lactates is significantly elevated. Therefore, to fully analyze the pRBCs, all or at minimum LAC and GLC (as presented in Fig. 2) parameters should be measured. At the same time, to increase the safety margin, any value out of range should serve as the basis for the rejection of the specific pRBCs (determining quality parameter). Each spectroscopic ratio depends on the concentration in SM of different chemical compound. In turn, concentration of these compounds in SM can vary on the donor profile^58^, therefore, each ratio should be compared with the appropriate RV-Exp or RV-Res values. The basis for the rejection of the specific pRBCs should be adjusted preferably on this ratio that is critical for the application (determining quality ratio).

We are presenting an example of analysis for chosen six pRBCs (donors A–F) carried out as illustrated in Fig. 2. We have compared the obtained values of LAC, GLC, HGB and LPD from RS measurements at each week of storage with the values of RV-Exp or RV-Res (Additional File 1: Figs. S11–S13). In case of the donor A the comparison of each ratio with RV-Exp suggests that the pRBCs should not be used after 6th week of storage. The comparison with RV-Res, based on LAC and GLC ratios, suggests that the pRBCs could be transfused to the patients with high risks of complication after transfusion, during first two weeks of the storage. However, while considering solely one ratio – HGB – only in the first week of storage the RV-Res quality conditions are met. Analysis of the additional LPD ratio, with 488 nm excitation (Additional File 1: Fig S13), indicates the use of pRBCs up two 2 weeks for restrict transfusion and almost up to 6 weeks until expiration time. In summary, based on the determining quality ratio, the analysis of pRBCs of donor A on the base of Raman spectrum obtained with 785 nm excitation, show that this the pRBCs from donor A could be transferred to the patients with high risks of complication after transfusion only in 1st week of storage while will expire for a standard use after the 6th week of storage. The same analysis was carried out for all donors A–F with the results presented Table 1.

**Table 1:** Summary of the results of the pRBCs analysis indicating the time period for exclusion in transfusion for the pRBCs (donors A-F) based on not fulfilling the proper reference values based on the label-free RS methodology (analysis of the LAC, GLC, HGB and LPD values presented in Additional File 1: Figs. S9–S11).

pRBCs DONOR	Age of pRBCs below which it could be transferred to the patients with high risks of complication after transfusion based on its quality [week of storage]	Age of pRBCs above which it is expired based on its quality [week of storage]
A	< 2	> 6
B	not enough quality even in the first week of storage	> 5
C	< 2	> 6
D	< 4	> 4
E	< 4	> 6
F	< 5	> 7

As presented in Table 1, based on spectroscopic evaluation, pRBCs of donor B had the worst quality from the beginning and should not be transferred to the patients with high risks of complication after transfusion. In addition the expiration based on its quality evaluation would be 5 weeks. Surprisingly pRBCs of donor D shows high quality until fourth week of storage followed by a rapid decline impacting significantly the expiry time (only 4 weeks). The best and longest quality is observed for pRBCs from donor F, that could be transferred to the patients with high risks of complication after transfusion until 5 weeks of storage and its quality maintains sufficient level for use even in 7th week of storage exceeding preset storage date. Our results clearly prove that the changes as well as their kinetics are different for each example of pRBCs, stressing the need for real time testing and indicating that our label-free strategy allows for the rapid, noninvasive, label-free evaluation of the biochemical state of pRBCs in real time at the point of use. The examples prove the applicability of presented strategy in determination of pRBCs quality for transfusion in clinical setting.

### Verification of the designed strategy in clinical environment

In the final stage of our project the pRBCs quality was analyzed with RS before the transfusion to AML or ALL patients (58 transfusions, N = 37). The RS measurement and the reference lactate quantification were carried out on the small sample of pRBCs (1 mL) obtained directly before transfusion. Subsequently, the pRBCs quality was assessed with the use of RS methodology and correlated with the feedback from the clinical setting based on patients’ condition before and after transfusion.

**Figure 3:**
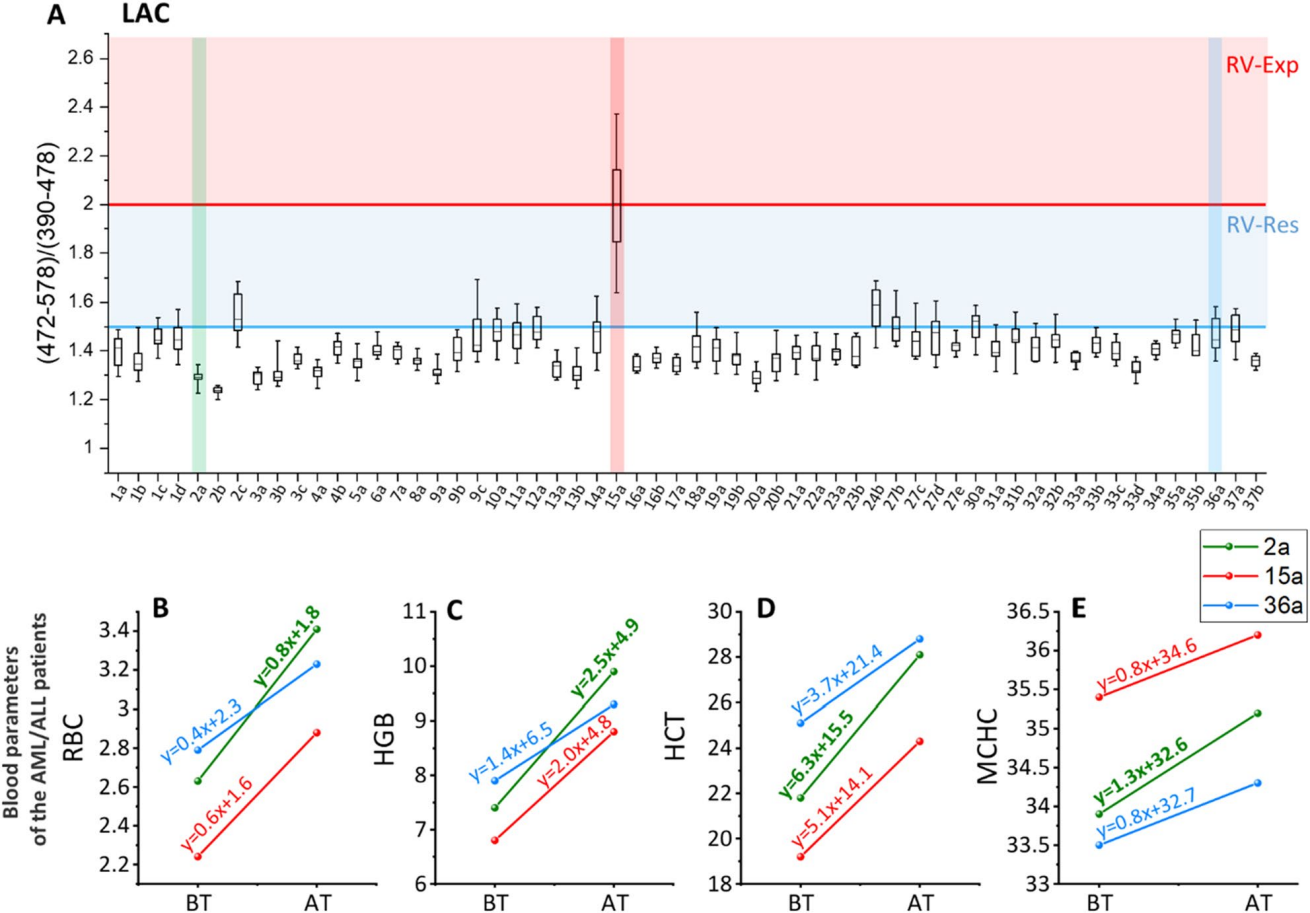
(**A**) LAC ratio values assessed using RS for pRBCs samples validated in clinical environment and intended for blood transfusion (58 transfusions, N = 37; letters a, b, c, d and e represent subsequent blood transfusion given to one patient – 1st, 2nd, 3rd, 4th and 5th respectively). The red region corresponds to pRBCs samples which do not comply the reference value of pRBCs expiration (RV-Exp, red area above RV-Exp line) and should not be considered for blood transfusion even for the healthy patients. The blue region corresponds to samples which do not meet the threshold value of pRBCs quality (RV-Res, blue area above RV-Res line) and can be considered for transfusion, however, should not be intended for transfusion to high-risk patients. Chosen blood parameters of the AML/ALL patients (HGB panel (**B**); HCT panel (**C**) and MCHC panel (**D**) recorded before (BT) and after (AT) the blood transfusion for the chosen samples (2a, 15a and 36a) showing less significant improvement of the blood parameters for the sample which do not meet RV-Res and RV-Exp values (blue and red line, respectively) comparing to the sample with optimal LAC ratio value (green line). The examples were only patients after first blood transfusion (marked with “a”) to eliminate influence of subsequent transfusions.

Figure 3A presents verification of RS method for pRBCs quality assessment performed on the pRBCs samples directly before their transfusion to AML or ALL patients (58 transfusions, N = 37), based on evaluation of the most restrict LAC ratio (for GLC ratio please see Additional File 1: Fig. S14). Procedure allowed for determination of pRBCs samples that do not meet RV-Res and RV-Exp thresholds, i.e. samples not intended for patients with an increased risk of post-transfusion complications or unsuitable for transfusion, respectively. Most of the studied pRBCs samples met the criteria of both, RV-Exp and RV-Rest, as expected since all pRBCs transfused in the clinics were no more than 2 weeks old. This procedure was developed to minimize the effect of older pRBCs on AML and ALL patients. During our study, while analyzing solely LAC ratio, one sample (15A) did not meet Rv-Exp value suggesting a high degradation of pRBCs (similar to values observed for expired pRBCs). Such pRBCs should not be considered for the blood transfusion even in healthy patients. In addition, even though pRBCs were stored no longer than 2 weeks, we have detected 12 pRBCs characterized by lower quality based on the restricted LAC ratio.

Subsequently, LAC ratio obtained from RS was correlated with patients’ health condition, monitored throughout the study before and after blood transfusion. According to the data presented in Additional File 1: Figs. S15 and S16, we observed increase in blood parameters for all studied patients after the blood transfusion. Unfortunately despite tracking complete blood count parameters (WBC, RBC, HGB, HCT, MCV, MCH, MCHC, PLT, etc.), reticulocytes, bilirubin, urea, creatinine, GFR, uric acid, LDH, CRP, complete lipidogram including cholesterol, HDL and LDL values, as well as analysis with advanced chemometrics including principal component analysis in a multivariate framework of all considered blood parameters, we did not find clear-cut correlation between spectroscopic quality evaluation and the short term patient response. The highest correlation coefficient was observed between LAC ratio obtained from RS with the absolute difference between the blood parameters recorded before and after the transfusion (Δ). The goal of this approach was to observe whether Δ grows with decreasing LAC ratio indicating the pRBCs samples with low LAC ratio are likely to provide less significant improvement of the blood parameters. Each studied blood parameters was investigated separately to find the relation using Spearman correlation coefficient. Its values oscillated between − 0.1 to 0.3. The lack of statistical significance of this correlation coefficient was proved using the appropriate statistical test, that delivered p-values much higher than commonly applied threshold α = 0.05. However, it must be stressed, that insignificance does not entail complete lack of correlation, but only means that we have not managed to find enough evidence to prove it being significant. The absence of the statistical significance in the noted deterioration in the rate of blood parameters improvement could be related to the significant individual diversity of patients receiving transfusions. The correlation between spectroscopic parameters and the patient condition remain obscure, as the blood transfusion efficiency do not only rely on the pRBCs quality itself, but also on the general health state of the patient undergoing the blood transfusion. In order to acquire the whole picture, the evaluation of long-term response of the patient's health condition would be beneficial. We have not carried it out in case of this method verification. It is however worth mentioning that the comparison of chosen samples that were spectroscopy defined as having the better quality (2a, much lower RV-Res value) with those of the worse quality (15a and 36a, close to RV-Exp and RV-Res, respectively) show the decrease in the rate of the improvement tracked by analysis of the blood parameters (HCT, HGB and MCHC, Fig. 3B–E). The rate of improvement was assessed by derivation of the linear equations and comparison of the slope values which in case of the sample 2a (green line) were always higher compared to the samples of lesser pRBCs quality (15a and 36a). Even though variations between 15 and 36a sample kinetic rates (as well as kinetic rates of other samples presented in the Additional File, Fig. S15) may be due to variety of a patient health condition before the blood transfusion^7,8,59^, i.e. various initial values of the considered blood parameter this observation highlights the strategy usefulness in assessment of pRBCs quality and confirms the validity of the applied Raman-based method.

## Supplementary Information

Supplementary Information.

## Data Availability

For original data please visit figshare dataset: https://doi.org/10.6084/m9.figshare.19153349.v1.
