# Supplementary material for: Label-free testing strategy to evaluate packed red blood cell quality before transfusion to leukemia patients
Source: Sci Rep. 2022 Dec 17;12:21849. doi: 10.1038/s41598-022-26309-5 (PMC9759565; doi:10.1038/s41598-022-26309-5)
Supplement: Supplementary file 1 — Supplementary Information. [file 41598_2022_26309_MOESM1_ESM.docx]

SUPPLEMENTARY INFORMATION

Label-free testing strategy to evaluate packed red blood cell quality before transfusion to leukemia patients

Jakub Dybas^a#^, Aleksandra Wajda^b#^, Fatih Celal Alcicek^a^, Magdalena Kaczmarska^a^, Katarzyna Bulat^a,c^, Ewa Szczesny-Malysiak^a^, Agnieszka Martyna^d^, David Perez-Guaita^e^, Tomasz Sacha^f^, Katarzyna M. Marzec^a,c*^

*^a^ Jagiellonian Center for Experimental Therapeutics, Jagiellonian University, 14 Bobrzyskiego St., 30-348 Krakow, Poland*

*^b^ Faculty of Chemistry, Jagiellonian University, 2 Gronostajowa St., 30-387 Krakow, Poland*

*^c^ Lukasiewicz Research Network, Krakow Institute of Technology, 73 Zakopiaska St., 30-418 Krakow, Poland*

*^d^ Forensic Chemistry Research Group, University of Silesia in Katowice, 9 Szkolna St., 40-006 Katowice, Poland*

*^e^ Department of Analytical Chemistry, University of Valancia, Dr. Moliner 50, Burjassot, Spain.*

*^f^ Department of Haematology, Jagiellonian University Hospital, 2 Jakubowskiego St., 30-688 Krakow, Poland*

^#^equally contributed

**Corresponding author**

Correspondence and material requests should be addressed to Katarzyna Maria Marzec, PhD, DSc.

Email: [katarzyna.marzec@kit.lukasiewicz.gov.pl](mailto:katarzyna.marzec@kit.lukasiewicz.gov.pl)

**Data sharing statement**

For original data please visit figshare Dataset: https://doi.org/10.6084/m9.figshare.19153349.v1


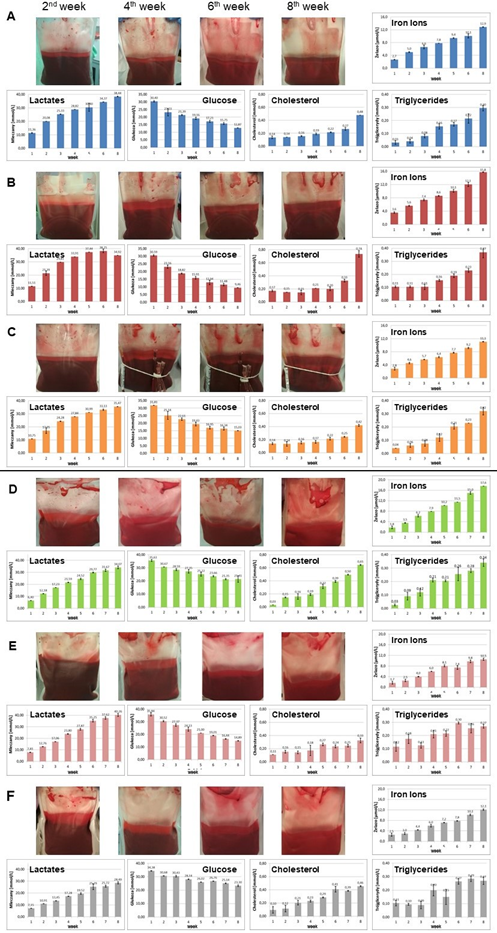


***Figure SI 1.****Progression of changes of three exemplary pRBCs (A–C) and three exemplary leukoreducedRBCs (D–F) during 56 days of their storage in the PVC storage bags in 4 °C. All presented biochemical data was collected using ABX Pentra 400 Analyser (Horiba Medical).*


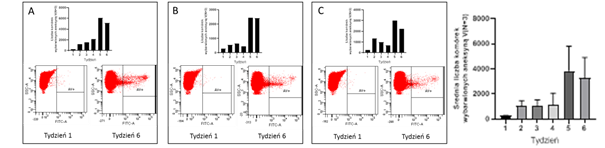


***Figure SI 2.****Flow cytometry analysis of apoptosis of human RBCs in exemplary pRBCs intended for transfusion as well as average results for N=27. RBCs stored in pRBCs were analysed every week with flow cytometry to assess information about their apoptosis. The analysis required staining with annexin-V labelled with fluorescein isothiocyanate.*


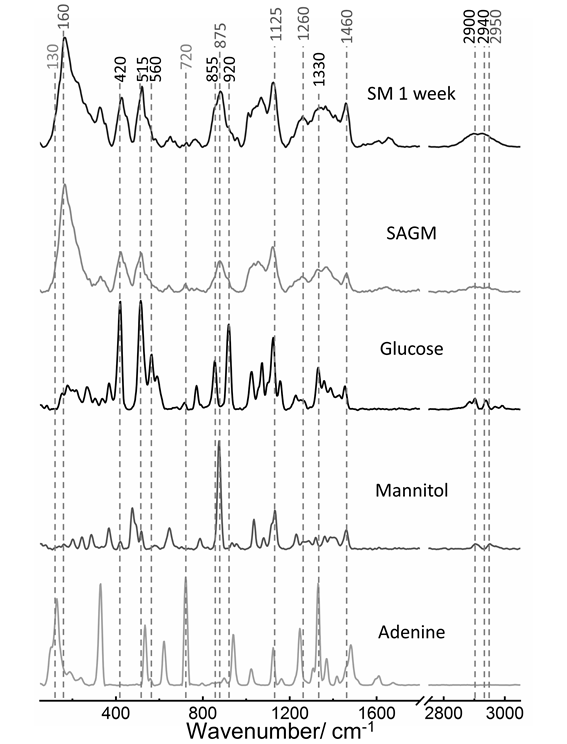


***Figure SI 3.****Average Raman spectra of the SAGM components: adenine (Sigma-Aldrich), mannitol (Sigma-Aldrich), glucose (D-glucose, Merck), SAGM (Maco Pharma) and exemplary Raman spectrum of leukoreduced RBCs after 1 week of storage. The spectra were recorded with 785 nm excitation wavelength, the laser power at the laser spot approximately 130 mW and are presented after baseline correction.*


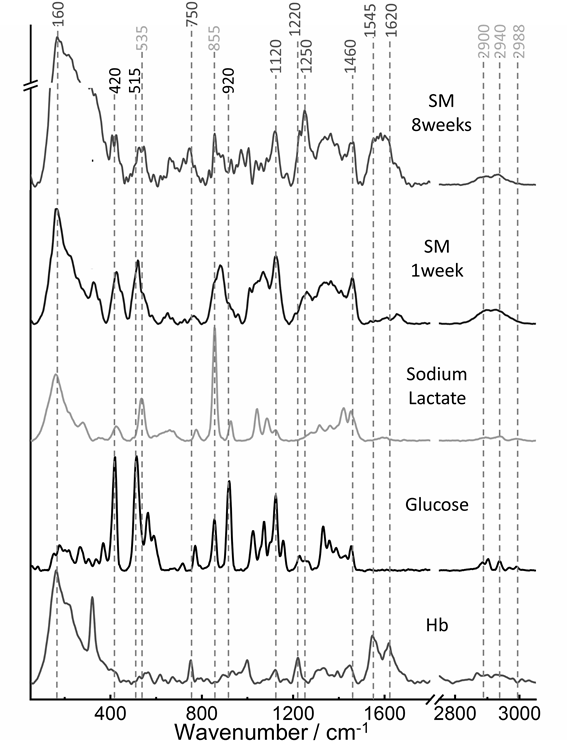


***Figure SI 4.****Average Raman spectra of the SM components: Hb, glucose (D-glucose, Merck), sodium lactate (Sigma-Aldrich) and exemplary Raman spectra of LRBCs after 1 and 8 weeks of storage. The spectra were recorded with 785 nm excitation wavelength, the laser power at the laser spot approximately 130 mW and are presented after baseline correction.*


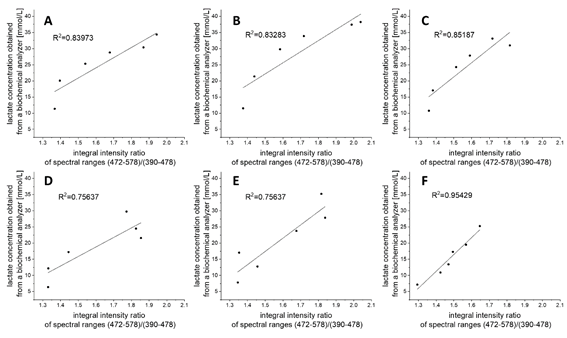


***Figure SI 5.****Correlation of the data obtained using RS and 785 nm excitation wavelength (the ratio of various lactic acid derivatives based on the integral intensities of the 472–578/390–478 cm^–1^ spectral ranges) with the reference data obtained using ABX Pentra 400 Analyser (concentration of lactic acid derivatives in mmol/L) within 6 weeks for three exemplary pRBCs (A–C) and three exemplary leukoreduced RBCs (D–F).*


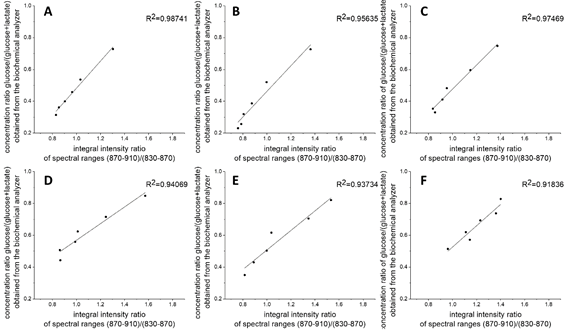


***Figure SI 6.****Correlation of the data obtained using RS and 785 nm excitation wavelength (the ratio of glucose content to the sum of glucose and lactic acid derivatives based on the integral intensities of the 870–910/830–870 cm^–1^ spectral ranges) with the reference data obtained using ABX Pentra 400 Analyser (the ratio of glucose concentration to the sum of glucose and lactic acid derivatives) within 6 weeks for three exemplary pRBCs (A–C) and three exemplary leukoreducedRBCs (D–F).*


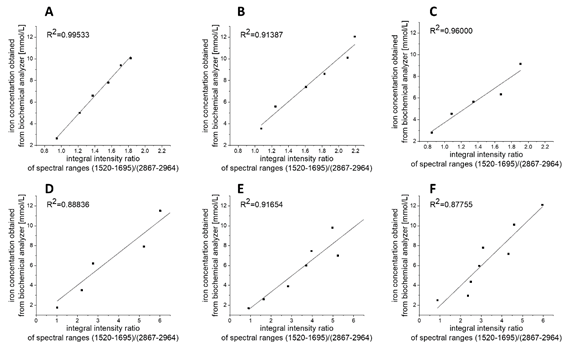


***Figure SI 7.****Correlation of the data obtained using RS and 785 nm excitation wavelength (the ratio of Hb content to the amount of C–H bonds present in all sample components based on the integral intensities of the 1520–1695/2867–2964 cm^–1^ spectral ranges) with the reference data obtained using ABX Pentra 400 Analyser (concentration of free iron ions in mmol/L) within 6 weeks for three exemplary pRBCs (A–C) and three exemplary leukoreduced RBCs (D–F).*


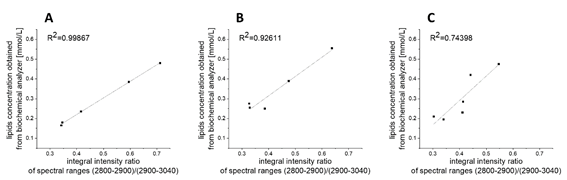


***Figure SI 8.****Correlation of the data obtained using RS and 488 nm excitation wavelength (the ratio of lipids to proteins based on the integral intensities of the 2800–2900/2900–3040 cm^–1^ spectral ranges) with the reference data obtained using ABX Pentra 400 Analyser (total lipid concentration in mmol/L) within 6 weeks weeks for three exemplary pRBCs (A–C).*

0

10

20

30

40

50

Actual (mM)

0

5

10

15

20

25

30

35

40

45

Predicted (mM)

**Glucose**

0

10

20

30

40

50

Actual (mM)

0

5

10

15

20

25

30

35

40

45

Predicted (mM)

**Lactate**

***Figure SI 9.****Predicted versus actual values for lactate and glucose PLSR. Blue and red circles indicate the independent prediction and cross validation, respectively. The green line represents the perfect prediction (1:1)*

400

600

800

1000

1200

1400

1600

Wavenumber (cm

-

1

)

10

12

14

16

18

20

22

24

Intensity

Glucose

Lactate

***Figure SI 10.****Regression vectors of the PLS models obtained for the prediction of lactate and glucose. Shaded boxes indicate the numerators (Blue) and denominators (red) of the ratios used as markers when evaluating the quality of blood.*


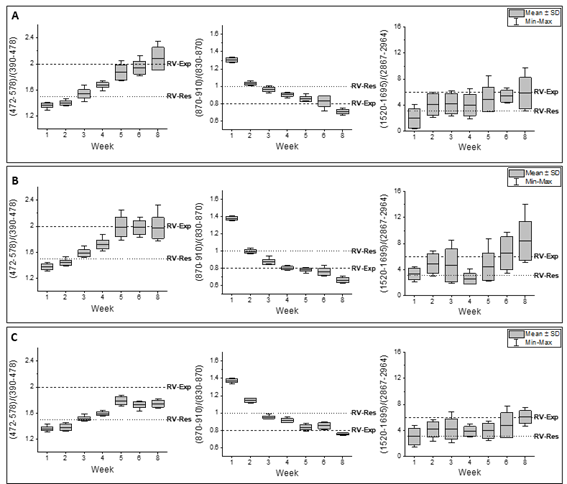


***Figure SI 11.****Set of box charts of the integral intensity ratios of metabolites found in the given SM samples obtained from the pRBCs based on the baseline-corrected Raman spectra recorded with 785 nm excitation wavelength (the ratios: 472–578/390–478 cm^–1^, 870–910/830–870 cm^–1^ and 1520–1695/2867–2964 cm^–1^ were acquired using A-type integration method). Box ends represent the standard deviation, horizontal line in the middle of the box represents the mean value and whiskers represent min and max values.*


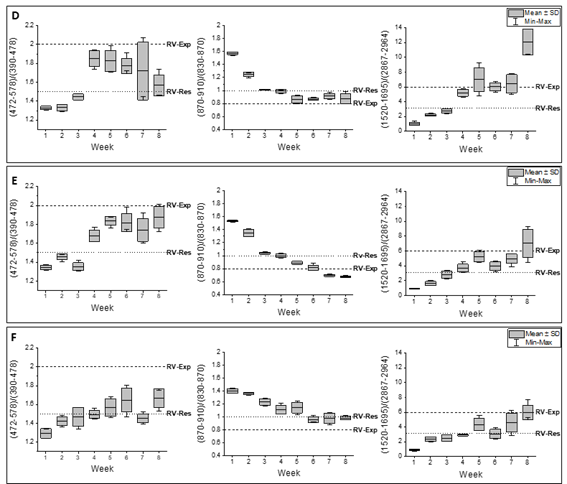


***Figure SI 12.****Set of box charts of the integral intensity ratios of metabolites found in the given SM samples obtained from the LRBCs based on the baseline-corrected Raman spectra recorded with 785 nm excitation wavelength (the ratios: 472–578/390–478 cm^–1^, 870–910/830–870 cm^–1^ and 1520–1695/2867–2964 cm^–1^ were acquired using A-type integration method). Box ends represent the standard deviation, horizontal line in the middle of the box represents the mean value and whiskers represent min and max values.*


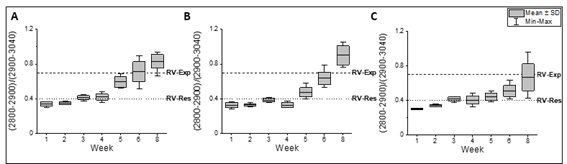


***Figure SI 13.****Set of box charts of the integral intensity ratios of metabolites found in the given SM samples obtained from the pRBCs based on the baseline-corrected Raman spectra recorded with 488 nm excitation wavelength (the ratio 2800–2900/2900–3040 cm^–1^ was acquired using A-type integration method). Box ends represent the standard deviation, horizontal line in the middle of the box represents the mean value and whiskers represent min and max values.*


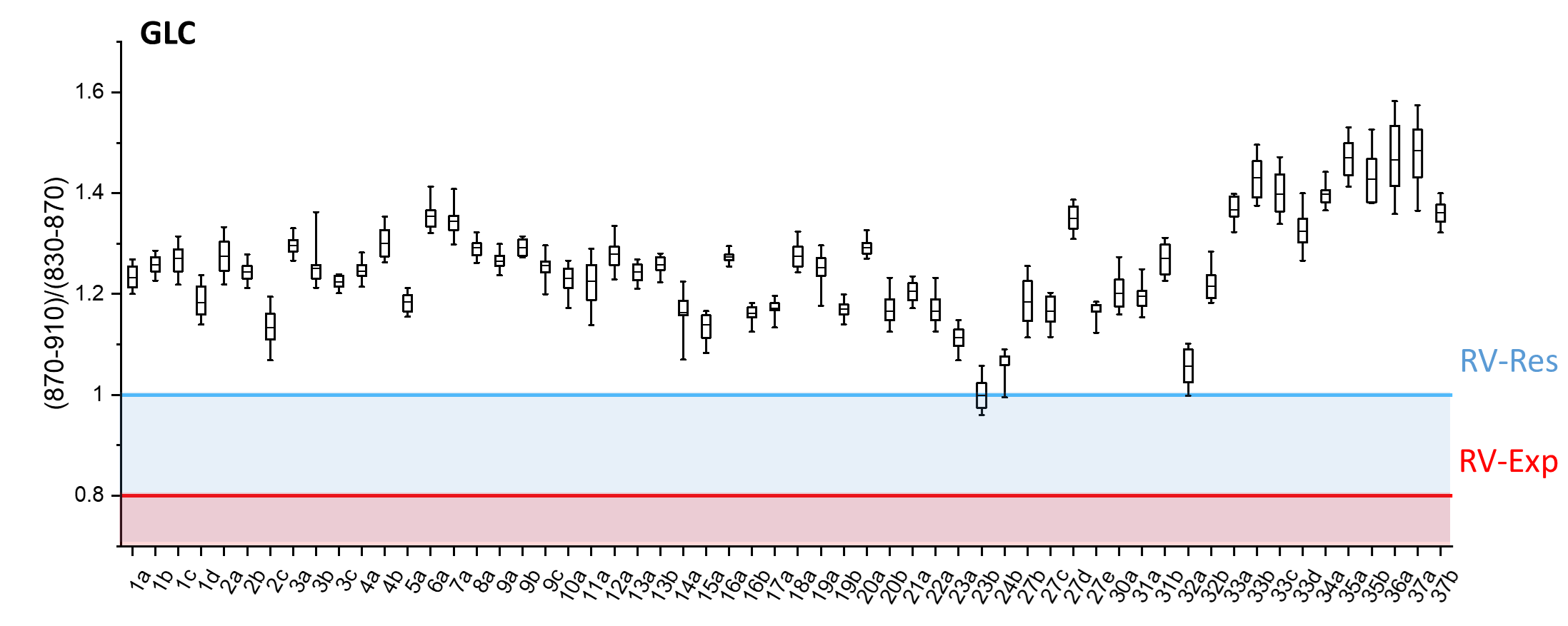


***Figure SI 14.****GLU ratio values assessed using RS for pRBC samples validated in clinical environment and intended for blood transfusion (58 transfusions, N=37). The red region corresponds to pRBC samples which do not comply the reference value of pRBCs expiration (RV-Exp, red area above RV-Exp line) and should not be considered for blood transfusion even in healthy patients. The blue region corresponds to samples which do not comply the restrict reference value of pRBCs quality (RV-Res, blue area above RV-Res line) and can be considered for transfusion, however, should not be intended for transfusion to high-risk patients.*


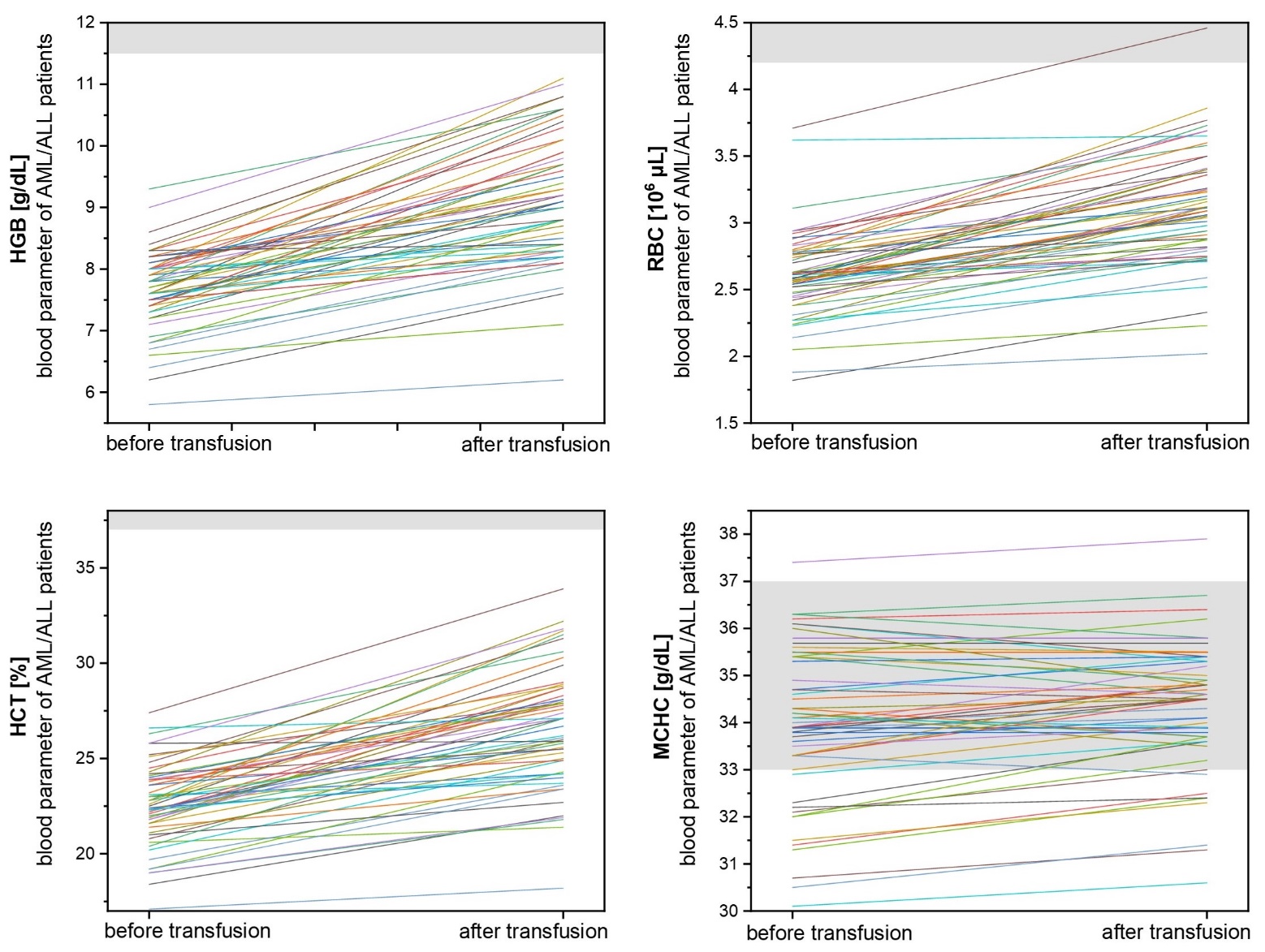


***Figure SI 15.*** *Variation in kinetics of the chosen blood parameters (HGB, RBC, HCT and MCHC) improvement for the AML/ALL patients acquired before and after the blood transfusion. In grey are marked areas corresponded to the normal ranges of the given blood parameter.*


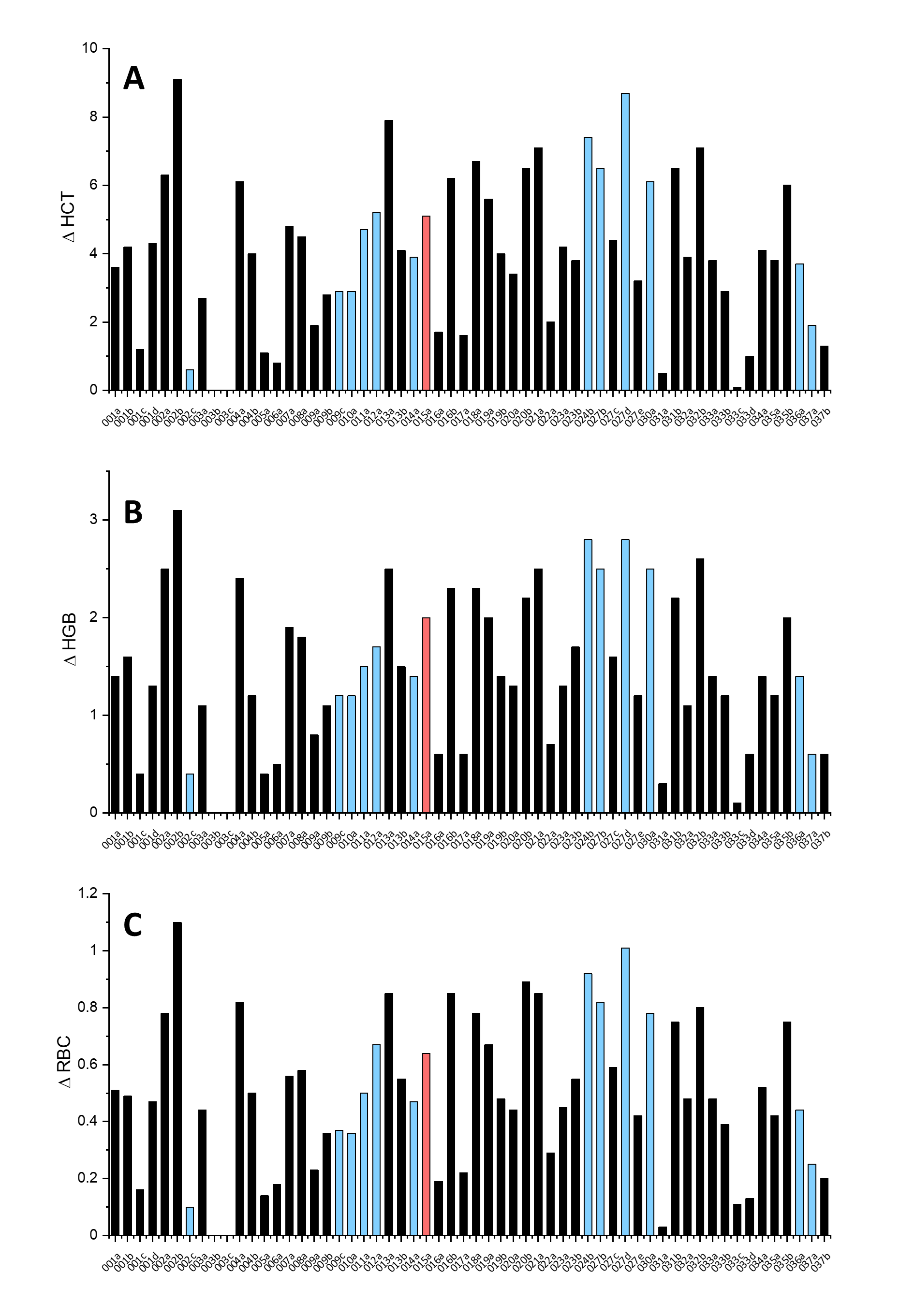


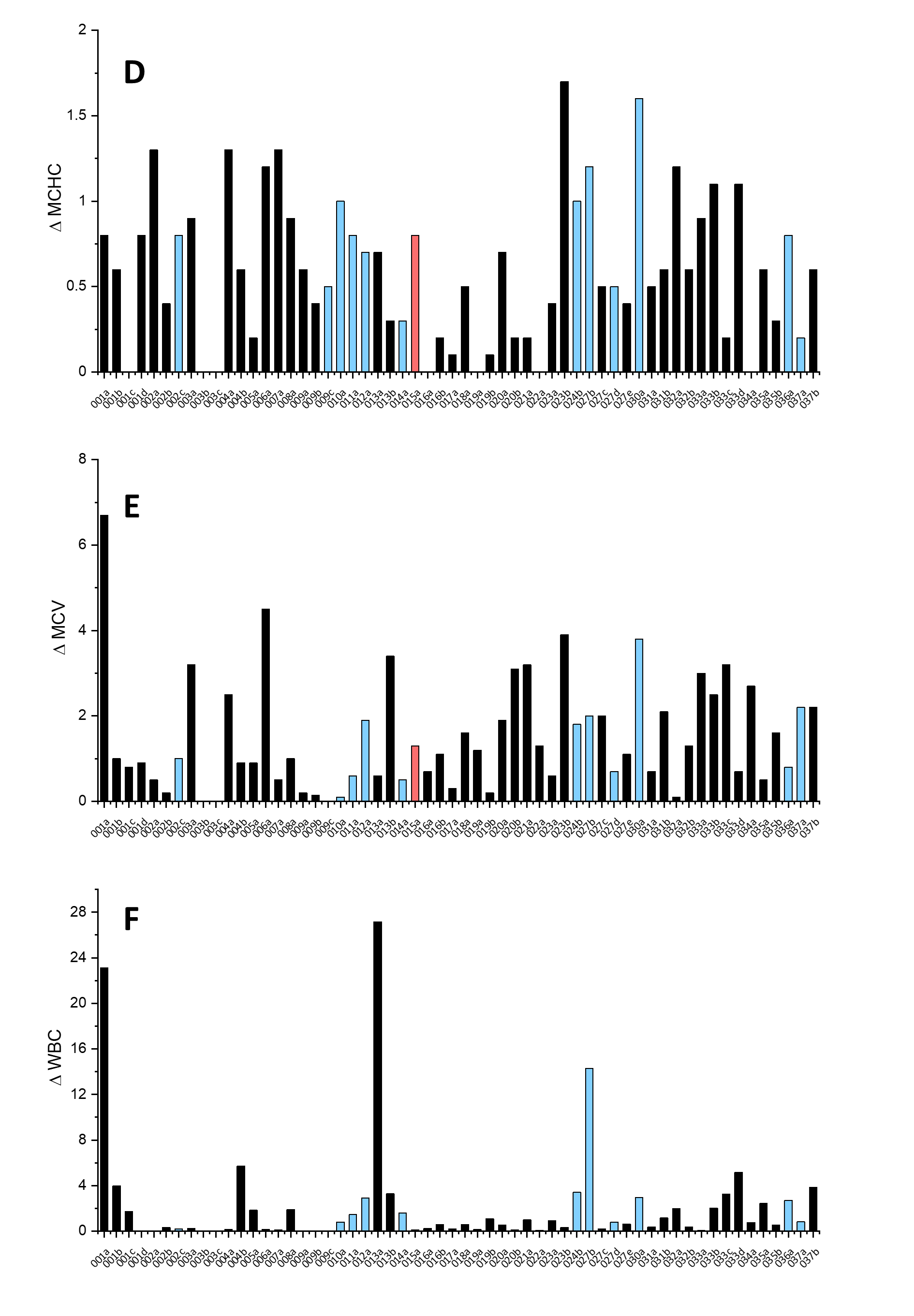


***Figure SI 16.****Variation in increment of the chosen blood parameter values (HCT, HGB, RBC and MCHC, MCV and WBC) for the AML/ALL patients after the blood transfusion. Blue and red bars correspond to the transfused pRBC samples which do not meet RV-Exp or RV-Res values, respectively.*

***Table SI 1***

*The band assignments for each spectral ranges observed in Raman spectra of the pRBCs and LRBCs after 1 and 8 weeks of storage. The band assignments of the Raman spectra were based on the measurements of the reference compounds with the use of 785 nm (a-h) or 488 nm (i) excitation wavelengths. The spectra are presented on the Figures 3 and 4.*

| **Spectral range** | **Band assignment** |
| --- | --- |
| <350 cm^-1^ | After 1 week of the pRBCs storage, band located at 160 cm^-1^ has similar profile to the SAGM spectrum. After 8 weeks of the pRBCs storage integral intensity of this band greatly increases what is connected with the increase in lactates concentration and amount of free Hb originated from RBC hemolysis. Therefore, increase in integral intensity of this band located in the spectral range 70–270 cm^–1^ with time of the pRBCs storage is connected with increases of lactates and free Hb concentrations with negligible decrease in adenine concentration (Sugita et al., 1965). Monitoring integral intensity of this band with time of the pRBCs storage corresponds to the increase in the concentration of both, lactates and glucose. Band located at around 323 cm^–1^ originate from vibrations in calcium fluoride, which in presented herein Examples is used as the substrate for the pRBCs and the reference compound measurements and was not considered in spectral analysis. |
| 390±3 – 478±3 cm^–1^ | Band located at around 420 cm^–1^ in the Raman spectra of the pRBCs after 1 week of storage corresponds mainly to the vibrations in glucose with negligible influence of mannitol. With the time of the pRBCs storage, impact of vibrations in glucose decreases while vibrations connected with the lactates presence increases (band at around 425 cm^–1^ originates from rocking vibrations of carboxylic ion, –COO^–^). Monitoring integral intensity of this band with time of the pRBCs storage corresponds to the decrease in glucose concentration accompanied by increase in lactates concertation. |
| 472±3 – 578±3 cm^–1^ | Band located at around 520 cm^–1^ in the Raman spectra of the pRBCs after 1 week of storage is connected with the presence of glucose and mannitol. With the time of the pRBCs storage, impact of vibrations in glucose decreases while vibrations connected with the lactates presence increases (band at around 535 cm^–1^ originates from wagging vibrations of carboxylate ion, –COO^–^). Monitoring integral intensity of this band with time of the pRBCs storage corresponds to the decrease in glucose concentration accompanied by increase in lactates concertation taking into consideration that mannitol concentration remains constant. |
| 700±3 – 790±3 cm^–1^ | Integral intensity of the band located in this range increases with the pRBCs storage as originates mainly from vibrations in Hb (750 cm^–1^) and lactates (775 cm^–1^). |
| 830±3 – 910±3 cm^–1^ | The broad band (with high full width at half maximum) located at around 880 cm^–1^ and observed in the pRBCs after 1 week of storage is combination of two bands originating from two glucose modes (855 and 920 cm^–1^) and mannitol mode (intense band at 880 cm^–1^). After 8 weeks of the pRBCs storage this band comprises also component from mode located at around 855 cm^–1^ connected with lactates presence. This band originates from stretching vibrations in carboxylate ion and corresponds to the overall contribution of all lactic acid derivatives (Cassanas et al., 1991). Monitoring integral intensity of the band at about 880 cm^–1^ with time of the pRBCs storage corresponds to the decrease in glucose concentration accompanied by increase in lactates concertation taking into consideration that mannitol concentration remains constant. Monitoring integral intensity of the band within 830–870 cm^–1^ spectral range with time of the pRBCs/LRBCs storage corresponds to the decrease in glucose concentration accompanied by increase in lactates concentration. Monitoring integral intensity of the band within 870–910 cm^–1^ spectral range corresponds mainly to the decrease in glucose concentration. |
| 970±3 – 1500±3 cm^–1^ | Comprises modes originated from SAGM components and changes within this spectral ranges corresponds mainly to the decrease in glucose concentration accompanied by increase in lactates concentration, however they remain obscure due to bands overlapping. The increase in integral intensity of band located at around 1260 cm^–1^ with time of the pRBCs/LRBCs storage corresponds to increasing glucose concentration. |
| 1520±3 – 1695±3 cm^–1^ | The broad band (with high full width at half maximum) located in this spectral range relates to the Hb appearance in SM with time of the pRBCs/LRBCs storage. Monitoring integral intensity of this band corresponds to the increase in Hb concentration in SM, and thus hemolysis level. |
| 2867±3 – 2964±3 cm^–1^ | Modes observed in this spectral range originate from C–H stretching vibrations. These bands are present in SAGM components, sodium lactate and Hb, and are typical for all biological components (Movasaghi et al., 2007). Monitoring integral intensity of bands in this spectral range corresponds to the overall content of all sample components. |
| 2800±3 – 3040±3 cm^–1^ | Bands located within the 2800 – 2900 cm^–1^ spectral range originate mainly from –CH_2_ stretching vibrations and are characteristic for lipid-related compounds, whereas bands located within the 2900 – 3040 cm^–1^ spectral range originate mainly from –CH_3_ stretching vibrations and are characteristic for protein-related compounds (Dybas et al., 2016; Movasaghi et al., 2007; Mo et al., 2009). Integral intensities of these bands are much higher on Raman spectra obtained with 488 nm excitation wavelength compared to 785 nm-excited Raman spectra due to the lower quantum efficiency of CCD detectors when excitation wavelength approaches near infrared (Krafft et al., 2016). Moreover, integral intensities of Raman bands depend on excitation wavelength (Czamara et al., 2015) and integral intensities of lipid-related bands increase with the excitation energy increase (i.e., excitation wavelength decrease) (Jamieson et al., 2018). Therefore, in order to assess lipids/proteins ratio it is preferably to use 488 or 532 nm excitation wavelength. Monitoring integral intensities of the bands within 2800–2900 cm^–1^ spectral range with time of the pRBCs/LRBCs storage corresponds mainly to the total lipid content. Monitoring integral intensities of the bands within 2900–3040 cm^–1^ spectral range corresponds mainly to the total protein content. |
